# Analyzing Diabetes Detection and Classification: A Bibliometric Review (2000–2023)

**DOI:** 10.3390/s24165346

**Published:** 2024-08-19

**Authors:** Jannatul Ferdaus, Esmay Azam Rochy, Uzzal Biswas, Jun Jiat Tiang, Abdullah-Al Nahid

**Affiliations:** 1 Electronics and Communication Engineering Discipline, Khulna University, Khulna 9208, Bangladesh; jebaferdaus@gmail.com (J.F.), esmayrochy@gmail.com (E.A.R.); 2 Centre for Wireless Technology (CWT), Faculty of Engineering, Multimedia University, Cyberjaya 63100, Malaysia

**Keywords:** diabetes detection, bibliometric analysis, word cloud, PRISMA

## Abstract

Bibliometric analysis is a rigorous method to analyze significant quantities of bibliometric data to assess their impact on a particular field. This study used bibliometric analysis to investigate the academic research on diabetes detection and classification from 2000 to 2023. The PRISMA 2020 framework was followed to identify, filter, and select relevant papers. This study used the Web of Science database to determine relevant publications concerning diabetes detection and classification using the keywords “diabetes detection”, “diabetes classification”, and “diabetes detection and classification”. A total of 863 publications were selected for analysis. The research applied two bibliometric techniques: performance analysis and science mapping. Various bibliometric parameters, including publication analysis, trend analysis, citation analysis, and networking analysis, were used to assess the performance of these articles. The analysis findings showed that India, China, and the United States are the top three countries with the highest number of publications and citations on diabetes detection and classification. The most frequently used keywords are machine learning, diabetic retinopathy, and deep learning. Additionally, the study identified “classification”, “diagnosis”, and “validation” as the prevailing topics for diabetes identification. This research contributes valuable insights into the academic landscape of diabetes detection and classification.

## 1. Introduction

Diabetes mellitus is a metabolic disorder characterized by prolonged hyperglycemia. As a chronic disease, diabetes causes a risk of developing various comorbidities, such as cardiovascular complications, hypertension, depression, thyroid gland disorders, chronic obstructive pulmonary disease (COPD), Alzheimer’s disease (AD), and retinopathy [1]. There are several types of diabetes, such as diabetic retinopathy, diabetic neuropathy, and diabetic foot ulcer. In 2021, the International Diabetes Federation recorded a worldwide adult diabetes prevalence of 536.6 million individuals, with projections suggesting an increase to 783.2 million by 2045 [2]. According to the International Diabetes Federation’s report, diabetes was among the top 10 global causes of death in 2021, responsible for 6.7 million fatalities [3]. Furthermore, the worldwide expenditure on medical costs associated with diabetes in 2019 reached USD 760 billion, with projections indicating an increase to USD 825 billion by 2030 and USD 845 billion by 2045. The financial strain imposed by diabetes-induced chronic diseases affects every nation [4].

Timely detection and prediction of diabetes are crucial for mitigating the risks associated with morbidity, hospitalization, and mortality. However, the conventional healthcare system’s protracted and expensive diagnostic processes have led to the integration of state-of-the-art technologies in diabetes prediction. Statistical analysis, machine learning (ML), and deep learning (DL) approaches are increasingly used in this domain. Global research on diabetes detection has experienced significant growth in recent decades, making it challenging for researchers to stay abreast of the latest findings. Simultaneously, understanding the future scope and knowledge gaps and identifying the factors receiving the most attention in diabetes detection are paramount.

Literature studies are practical tools to address these challenges, offering overviews of existing research on specific topics. Among the various techniques available for literature review, we opted for bibliometric analysis in this study, due to its ability to provide quantitative information concisely. Bibliometric analysis is valuable as it uses mathematical and statistical techniques to analyze, track, and evaluate publications’ quantitative connections and impacts in a specific field. This method aids in identifying significant research, scholars, journals, institutions, and countries within a defined period, offering a quantitative overview of the extensive academic literature. The choice of bibliometric analysis is supported by its successful application in diverse biomedical fields, including hypertension [5], cancer [6], arrhythmia [7], heart disease [8], and stroke [9]. Numerous researchers have used bibliometric analysis to explore diabetes, highlighting its effectiveness in uncovering trends, key contributors, and advancements.

In 2022, Zhang et al. [10] conducted a bibliometric diabetes analysis. The study identified 3029 papers published from 2000 to 2020 using the Web of Science Core Collection. The United States emerged as the most prolific country, contributing 965 papers, while the University of Alberta stood out as the leading institution, with 76 publications. *Diabetes Care* emerged as the top journal, publishing 178 papers and being the most co-cited (2630 times). Among authors, Riddell MC [11] had the highest number of publications (53), while Sigal RJ [12] emerged as the most influential author, with 503 citations. Colberg et al.’s paper exhibited the most substantial citation bursts by the end of 2020, making it a representative reference with 183 co-citation counts. The study identified four leading research studies on diabetes mellitus, exercise, physical activity, and glycemic control. Two prominent frontier trends were highlighted: sedentary behavior and stress. Conversely, our research especially focuses on diabetes detection and classification research from 2020 to 2023. Using the PRISMA 2020 framework analyzing 863 publications, this study identifies India, China, and the United States as leading countries. It highlights keywords like machine learning, diabetic retinopathy, and deep learning, with prevailing topics being classification, diagnosis, and validation. Methodologically, while both studies use performance analysis and science mapping, our research follows a more structured approach, with the PRISMA 2020 framework helping in the identification and selection of relevant papers. Zhang et al. [10] explored a broader view of research impact, whereas our research provides a specific advancement in diabetes detection and classification. So, both studies reflect different representations and trends.

Subramanian et al. [13] used bibliometric analysis on diabetes retinopathy detection. For this purpose, they conducted comprehensive data analysis, focusing on various aspects, such as publications, leading countries, primary sources, subject areas, prominent authors, emerging topics, co-occurrences, thematic evolution, factorial mapping, and citation analysis. Zheng et al. [14] performed bibliometric analysis on diabetic peripheral neuropathy pain. They used 1422 articles that were published from 2011 to 2021. According to their analysis, the United States had the leading position in this field and collaborated with Italy, Japan, and other countries. Pfizer stood out with the most significant number of publications, totaling 53. Tesfaye S [15] emerged as the most prolific author regarding study contributions.

This study is structured into five primary sections. Section 1 is an introduction. Section 2 outlines our proposed methodology for conducting bibliometric analysis. Section 3 has discussed prominent authors, institutions, countries, emerging trends, and novel themes in diabetes detection and classification. Section 4 presents various forms of network analysis. Finally, Section 5 and Section 6 encapsulate our findings and conclude our research.

## 2. Materials and Methods

**Figure 1:**
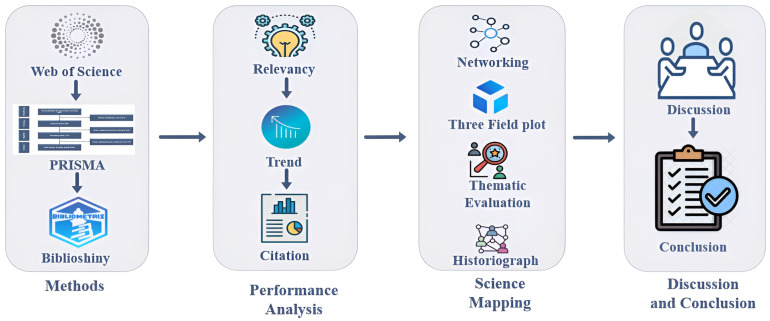
Proposed methodology for bibliometric analysis.

This study conducted a bibliometric analysis focusing on diabetes detection and classification. This method provides a comprehensive overview of the academic literature and facilitates identifying influential research, authors, journals, institutions, and countries over time. With the significant advancements in scientific technology, various bibliometric methods and tools have emerged to aid researchers in their investigations. This rigorous quantitative approach entails analyzing the interconnections and impacts of publications within a specific research field using statistical and mathematical techniques. In 1934, Paul Otlet first used bibliometrics in his book *Traite de Documentation* [16]. From among the different databases, we used the Web of Science database, which contains a significant amount of relative resources [10]. We performed our work in different parts, such as topic selection, searching tool, PRISMA, data mining tool, and performance analysis. Figure 1 illustrates the steps of our workflow.

### 2.1. Data Finding Using the PRISMA Framework

We chose “diabetes detection and classification” as our focal topic for our bibliometric analysis. Despite considerable prior research in this area, we selected this topic to investigate its historical evolution and present status in depth. Through this study, we aimed to identify the key contributors—authors, institutions, journals, and community members—in diabetes detection and classification, shedding light on their significant contributions.

**Figure 2:**
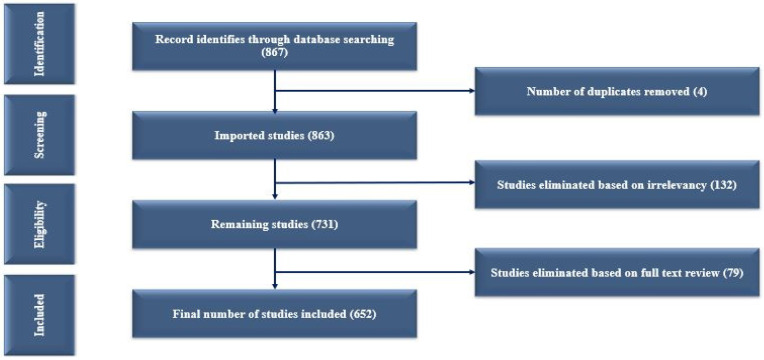
PRISMA flow diagram.

The first step in our work was “data finding using the PRISMA framework”. PRISMA is a four-phase workflow that involves identifying, selecting, and excluding documents, with reasons for exclusions provided. The PRISMA 2020 framework illustrates how information flows across the different phases of a bibliometric review (Figure 2). Many search tools, such as the Web of Science (WoS), Scopus, and PubMed, are available to collect relevant papers on specific topics. We used the WoS as a search tool for this study as we did not have academic access to Scopus. We searched the WoS database for “diabetes detection” and “diabetes classification” and combined the two keywords into “diabetes detection and classification”. In 2000–2023, the Web of Science revealed 867 papers on “diabetes detection and classification”.

We found 867 papers on diabetes detection and classification throughout this time. These papers were not directly related to our research, so we followed the principle of PRISMA. The PRISMA model was first published by Moher et al. [17]. The PRISMA flow diagram represents how many paper were identified, selected, and discarded. Of the initial 867 papers, we identified 4 duplicates, resulting in 863 unique papers being imported. Subsequently, 132 irrelevant papers were excluded from the screening section. In our study, we only included those papers whose titles contained the words “diabetes detection and classification”, along with the terms “machine learning” and “deep learning”. We tried to ensure that our research excluded those papers that focused on public issues or different diseases. We excluded these 132 papers by manual screening as the papers failed to meet our predefined selection criteria. Next, 731 papers were further analyzed. Within this set, 79 papers were classified as review articles. Finally, using the PRISMA method for bibliometric analysis, we included 652 papers for comprehensive examination in the journal.

### 2.2. Data Mining Tool

Selected papers can be effectively analyzed using various data mining tools, including Biblioshiny, VOSviewer, Gephi, Histcite, and citeSpace. For this research, we selected Biblioshiny software (version 4.1) to explore, evaluate, and develop the graphical visualization. M. Aria and C. Cuccuullo developed the Biblioshiny tool [18], an R statistical programming language tool especially designed for quantitative assessment. Biblioshiny offers visualization options like line plots, bar graphs, tables, and maps, which aid researchers in conducting their studies. Furthermore, it can be integrated with other data mining software, including Excel and R. All analyses for this study were conducted using Biblioshiny software.

### 2.3. Performance Analysis

To assess research performance, growth and scientific trends in diabetes detection and classification, we used various bibliometric attributes, including citation analysis, trend analysis, and network analysis. Our analysis considered multiple bibliometric dimensions, including author, journal, and institutional performance. This paper presented two types of bibliometric analyses. Firstly, we conducted a performance analysis, focusing on individual, group, or organizational contributions to the research topic. Secondly, we focused on science mapping, emphasizing the visualization of relationships and interconnections among authors, journals, and institutions. Performance analysis aims to identify significant authors, sources, countries, or affiliations within the research field. At the same time, science mapping helps uncover historical development, gaps in the literature, and emerging or declining research trends. In the subsequent sections, we explain performance analysis and science mapping by examining the 652 selected papers.

## 3. Bibliometric Performance Analysis for Diabetes Detection and Classification

Bibliometric performance analysis observes the researcher’s contribution in a specific field and investigates the overall progress in that field. In this section, we analyzed various bibliometric performance indicators, such as the publication number, trends, and citations. This exhibited a comprehensive understanding of diabetes detection and classification from 2000 to 2023.

### 3.1. Leading Countries, Authors, Affiliations, and Sources Based on the Number of Publications

The countries, authors, affiliations, and sources that most contributed to the field are illustrated in this section based on the number of their publications.

#### 3.1.1. Most Productive Countries

**Figure 3:**
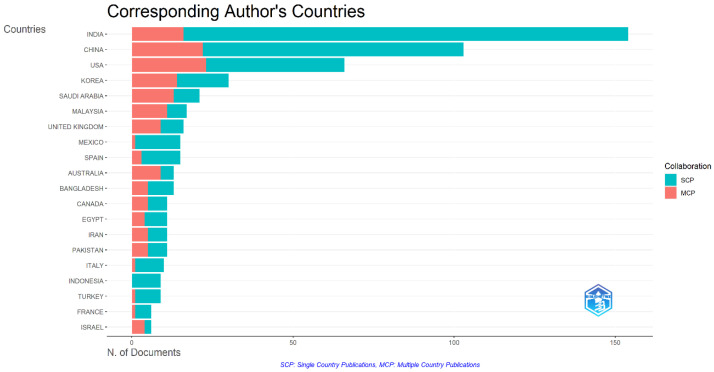
The most productive countries.

Figure 3 represents the 20 most productive countries or the corresponding author’s country for diabetes detection and classification. These countries have produced the highest number of publications in terms of diabetes detection and classification. The orange box indicates multiple-country production, and the blue box indicates single-country production. These document plots represent both single-country production and multiple-country production. Here, India and China outperformed all other countries. According to this analysis, India was the most productive country, with almost 150 or more documents on diabetes detection and classification. China was the second-most productive country, having published nearly 100 or more articles on diabetes. At the same time, the United States was in third position, having about 75 documents. The Republic of Korea and Saudi Arabia posted 45 and 30 articles, respectively, on diabetes detection and classification. The last 10 countries had 20 to 10 articles, far fewer than India and China. From Figure 3, we can conclude that India contributes more to diabetes detection research, and China has shown more domination in this area.

#### 3.1.2. Most Relevant Authors

**Figure 4:**
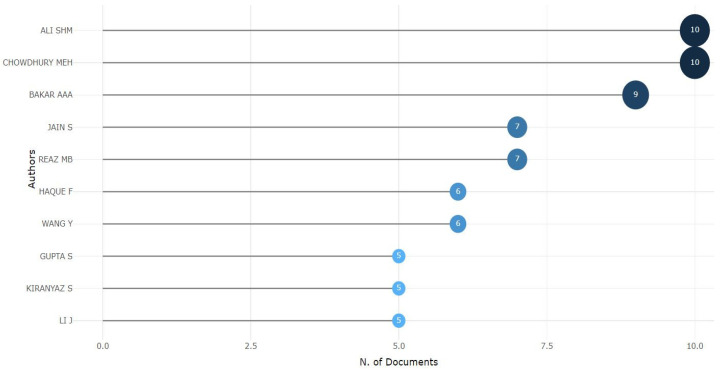
The most relevant authors.

Many authors and research institutes have extensively explored the study area of diabetes detection. In this section, we look into the authors contributing to diabetes detection, as shown in Figure 4. One author’s work can entirely focus on a fixed field or explore different areas of interest to solve problems. Figure 4 shows each researcher’s dedicated work regarding their number of publications. It indicates that ALI SHM [19,20,21] and CHOWDHURY MEH [22,23] had 10 publications each. Next, we had BAKAR AAA [22], with nine publications, and JAIN S [24] and REAZ MB, with seven publications each. Next, we had six publications each by HAQUE F and WANG Y. Positions 8, 9, and 10 were acquired by GUPTA S [25], KIRANYAZ S, and LI J [26], who had 5 publications each. Understanding the most relevant authors in the field of diabetes detection will help us stay up to date on developments by authoritative figures.

#### 3.1.3. Most Relevant Affiliations

**Figure 5:**
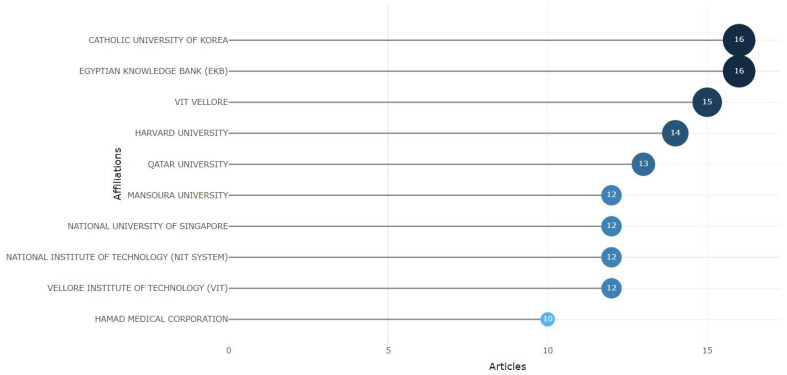
The most relevant affiliations.

The output of this analysis was the most relevant affiliations regarding diabetes detection by different institutes. The most pertinent affiliation indicates how one institute has excelled in that particular research area or topic. It may be because of research expertise or because these institutes can specialize. Figure 5 shows that the highest affiliation number is 16, achieved by the CATHOLIC UNIVERSITY OF KOREA and the EGYPTIAN KNOWLEDGE BANK. Next, we have VIT VELLORE, which has 15 affiliations, and HARVARD UNIVERSITY, which has 14 affiliations. Subsequently, we have QATAR UNIVERSITY, which has 13 affiliations. The following institutes have achieved 12 affiliations each: MANSOURA UNIVERSITY, the NATIONAL UNIVERSITY OF SINGAPORE, the NATONAL INSTITUTE OF TECHNOLOGY (NIT SYSTEM), and VELLORE INSTITUTE TECHNOLOGY (VIT). Finally, we have HAMAD MEDICAL CORPORATION, with 10 affiliations. This affiliation number indicates that several institutes are active in interdisciplinary collaboration in diabetes detection.

#### 3.1.4. Most Relevant Sources

**Figure 6:**
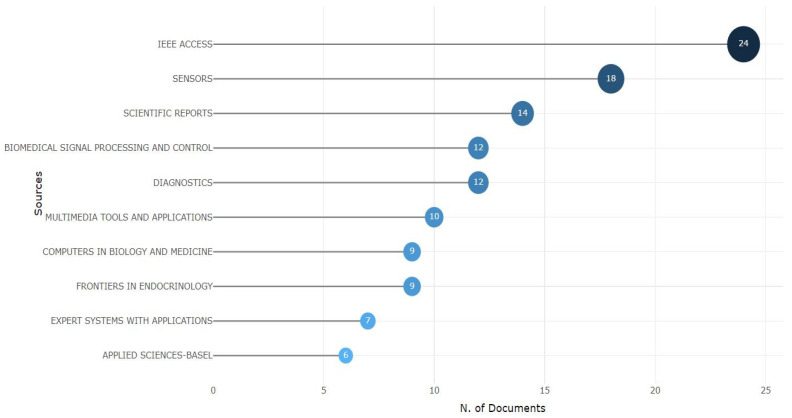
The most relevant sources.

In this section of bibliometric analysis, we look into the most relevant sources of publishers in diabetes. The most relevant sources indicate how the journal is empowering one of the specific research areas of the field. The top 10 journals shown in Figure 6 primarily publish topics on diabetes detection. From Figure 6, we can see *IEEE Access* has been number 1 in this field, with 24 publications. *SENSORS* secured the second place, with 18 publications, and *Scientific Reports* is in position three, with 14 journals. *Biomedical Signal Processing and Control*, *Diagnostics*, *Multimedia Tools and Applications*, and *Computers in Biology and Medicine* sequentially follow in the next positions. *Applied Sciences Basel* secured the 10th position, with six published journals on diabetes detection. In short, this most relevant source indicates how recent journals have emphasized diabetes detection and contributed sufficient publications in this domain.

### 3.2. Trend Analysis

Trend analysis involves trending topics in specific research areas over a particular period. Trend analysis is a practical analysis in academic research and provides a relationship between different variables in the data.

#### 3.2.1. Word Cloud of Keywords

**Figure 7:**
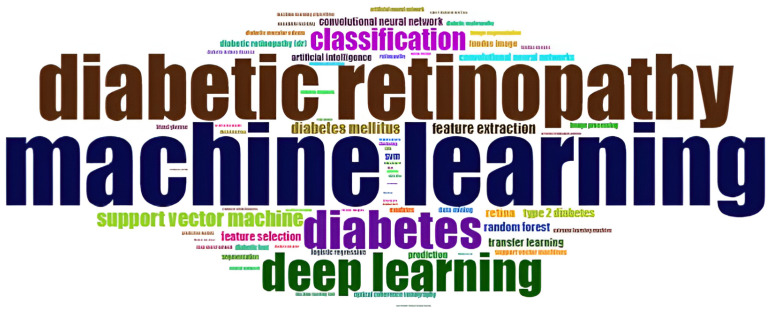
Word cloud of keywords.

Figure 7 depicts the 50 most frequently used keywords in the articles. Here, the frequency of each word is represented by its size. The most frequently used words in the diabetes detection and classification research area were “machine learning” and “diabetic retinopathy”. “Deep learning”, “diabetes”, and “classification” were the second-most frequent words, which had less physical dimension than “machine learning” and “diabetic retinopathy”. Other available keywords in this cloud were not used much, and their performance rate was much less.

#### 3.2.2. Growth of Top 10 Keywords of Authors

**Figure 8:**
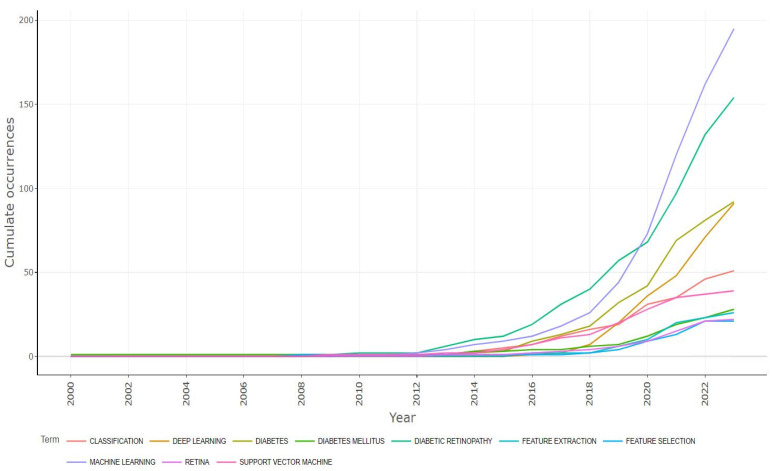
An outgrowth of top 10 keywords.

Figure 8 represents the outgrowth of the top 10 keywords of authors in the diabetes detection and classification research area. The keywords were “classification”, “deep learning”, “diabetes”, “diabetes mellitus”, “diabetes retinopathy”, “feature selection”, “feature extraction”, “machine learning”, “retina”, and “support vector machine”. Each keyword has an annual growth; researchers should be conscious of this continuous development in research areas. In Figure 8, the *Y*-axis indicates the cumulative occurrences, and the *X*-axis indicates the year. The keyword “machine learning” had an excellent performance curve in the diabetes detection and classification research area. The term “machine learning” first appeared in 2000, and by the end of 2022, its cumulative occurrence number had reached 190. Similarly, the term “diabetes mellitus” was first used in 2000; by the end of 2022, its growth rate had reached 160. Among the 10 keywords, “machine learning” first attracted the authors’ attention. And the third- and fourth-highest keywords were “diabetes” and “deep learning”, respectively. Their cumulative occurrence numbers were 80 and 78. After that, the rest of the keywords had almost the same cumulative occurrence rates.

#### 3.2.3. Trending Topics

**Figure 9:**
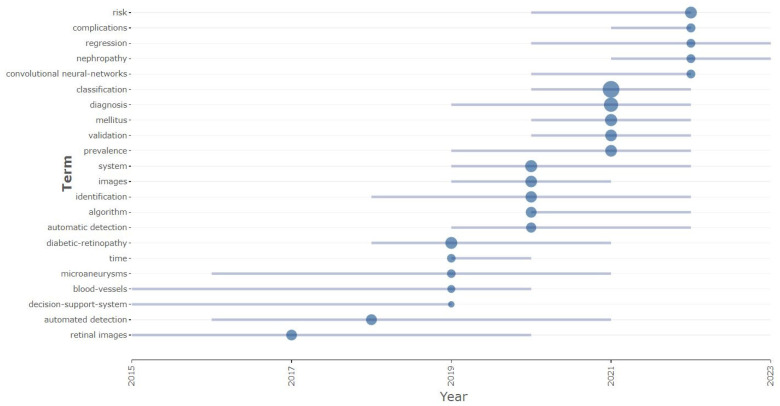
Trending topics.

Trending topics reveal people’s preferences, as well as what is occurring currently. It is a handy tool for researchers. Figure 9 represents the trending topics from 2015 to 2023 based on their use in diabetes detection and classification research. Here, the smallest circle represents the minimum term frequency of 25, the middle circle represents the medium term frequency of 50, and the largest circle represents the higher term frequency of 75. At the beginning of diabetes research, “decision support system” was the most trending topic from 2015 to 2019. From 2020 to 2022, some of the topics were hot topics. These were “diabetes mellitus”, “classification”, “diagnosis”, and “validation”. Among them, “classification” had the highest frequency, which is 75. Also, we could predict that “regression” and “diabetes nephropathy” would be on the trend because these two topics from 2020 and 2021, respectively, would still be visible in 2023.

### 3.3. Citation Analysis

Citation analysis is conducted based on how frequently a work is cited. Also, the work is analyzed by observing where, by whom, and in which context it is cited. Citation analysis evaluates the quality of research works. Using this analysis, we investigated the most influential authors and sources.

#### 3.3.1. Most Cited Authors

**Figure 10:**
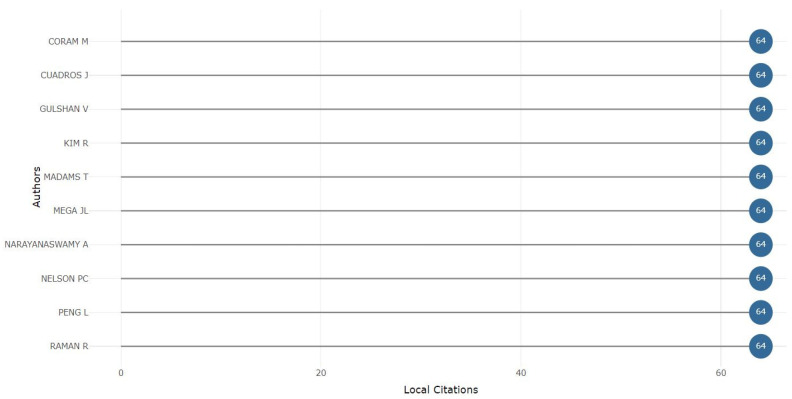
Most locally cited authors.

The output of this analysis indicated the most locally cited author in diabetes detection, which provided information about their citation frequency, collaboration, and work significance in the local research community. Figure 10 shows the authors CORAM M, CUADROS J, GULSHAN V, KIM R, MADAMS T, MEGA JL, NARAYANSWAMY A, NELSON PC, PENG L, and RAMAN R [27,28,29,30] all had an equal number of citations, which is 64. The citation number indicated their research focus was different in diabetes detection. Because of their versatile work, each author had many citation records. The consistent local citations of these authors showed that this locally cited author number is vital for measuring the impact of regional research.

#### 3.3.2. Most Cited Sources

**Figure 11:**
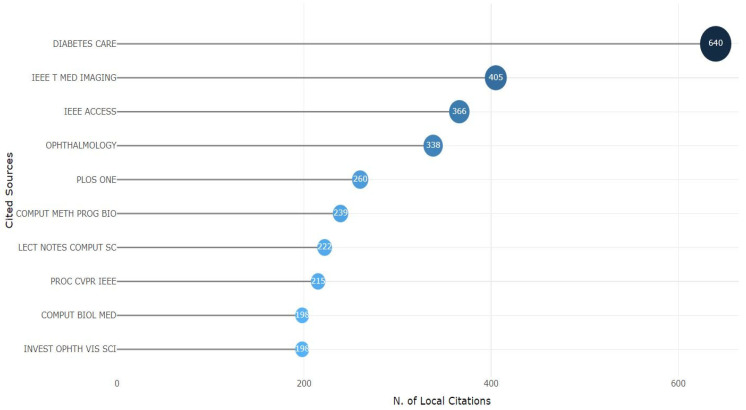
Most locally cited sources.

This section assesses the most locally cited sources in diabetes detection. Local citations indicate references from the same research community. They also help to exchange information and quotations within the same geographic or institutional setup. From Figure 11, we can see that *DIABETES CARE* had the highest number of citations, 640. Next, we had *IEEE T MED IMAGING*, with a citation number of 405. After that, we had *IEEE ACCESS*, *OPHTHALMOLOGY*, *PLOS ONE*, *COMPUT METH PROG BIO*, *LECT NOTES COMPUT SC*, and *PROC CVPR IEEE*, with a citation number of 366, 338, 260, 239, 222, and 215, respectively. At the end of the list, we had *COMPUT BIOL MED* and *INVEST OPHTH VIS SCI*, with a citation number of 198 each. Local citation numbers showed that they have an internal citation of significant collaboration with diabetes detection, which seems promising as it offers substantial knowledge sharing between the local community.

## 4. Science Mapping

Science mapping visualizes the connection between different research domains and scientific concepts. It is a kind of network diagram. This diagram consists of additional analyses.

### 4.1. Network Analysis

Network analysis analyzes the relationship between various journals, countries, and affiliations. It helps researchers to know about collaborative learning. This analysis plays a vital role in the progress of a research field.

### 4.2. Collaboration Network of Countries

**Figure 12:**
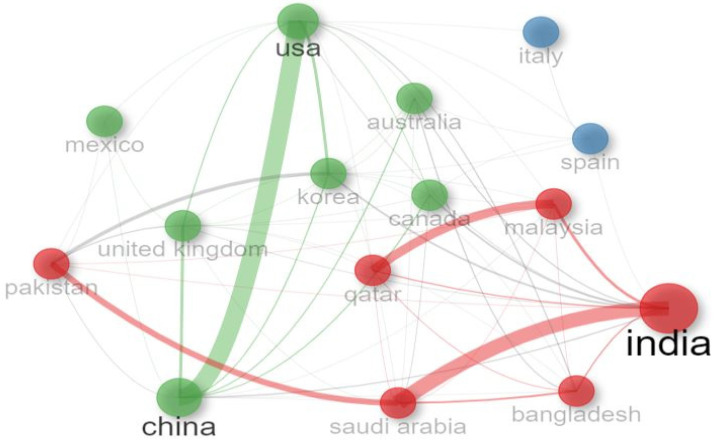
Collaboration network of countries.

Figure 12 indicates the collaboration network of countries working on diabetes detection and classification. There are three types of color clusters (red, green, and blue), and the line between them of the same color indicates the collaboration country. The thicker the line, the more the collaboration between those countries for diabetes research. Figure 12 shows that the United States and China had the highest collaboration, and then we had India and Saudi Arabia. In addition, the circle size of the cluster indicates the collaboration frequency. In this case, India collaborated more closely than the United States and China. The green cluster has the highest number of countries, which means the United States, China, the Republic of Korea, Australia, Canada, Mexico, and the United Kingdom had been working together to classify and detect diabetes. Next, we have a red cluster, which includes India, Bangladesh, Saudi Arabia, Qatar, Malaysia, and Pakistan. The smallest cluster size is blue, which includes Italy and Spain. It was concluded that the most significant cluster network was the green cluster, which had the highest number of collaborative countries.

### 4.3. Co-Citation Network of Journals

**Figure 13:**
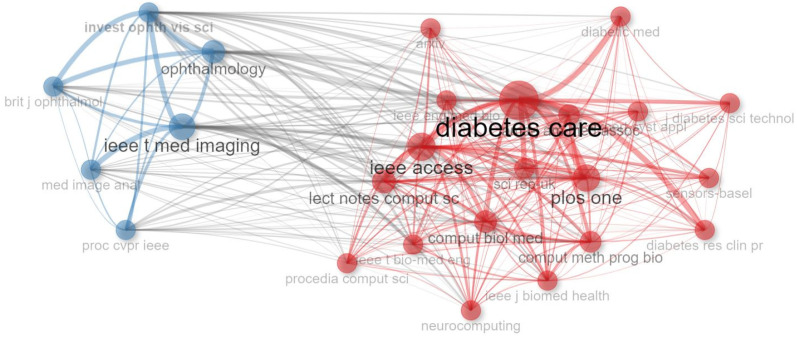
Co-citation network of journals.

A co-citation network of journals indicates a relationship between journals cited in the same article. Figure 13 contains the names of 25 journals. It has two different clusters: blue and red. The red cluster indicates biological and computational journals, and the blue cluster indicates ML base journals. Both the clusters include diabetes detection and classification journals. We can see from the blue cluster that *IEEE T Med Imaging*, *Ophthalmology*, *Med Image Anat*, etc., cited each other’s papers in their articles. On the other handHowever, in the red cluster, *Diabetes Care*, *PLOS One*, *IEEE Access*, *Neurocomputing*, and many others cited each other’s papers in their articles. Analyzing this figure, we can conclude that there exists a strong relationship between the journals named *Diabetes Care*, *IEEE Access*, *PLOS One*, and *Lecture Notes in Computer Science* (*Lect Notes Comput Sc*).

### 4.4. Collaboration Network of Institutions

**Figure 14:**
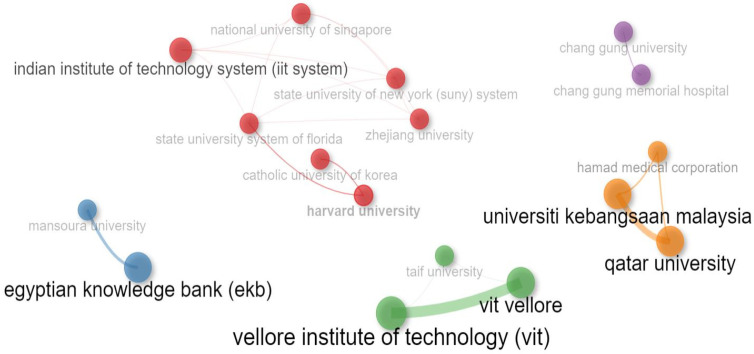
Collaboration network of institutions.

Figure 14 illustrates the collaboration network among institutions in the diabetes detection and classification research field. Each institution is represented by a colored circle, with the size of the circle indicating the publication frequency of that institution. Lines of the same color denote collaboration between institutions. The network revealed five clusters, distinguished by different colors: red, blue, green, yellow, and purple. Institutions within each cluster collaborated on research activities irrespective of geographical proximity. The red cluster is the largest, comprising seven universities from diverse geographical regions, such as India, the United States, Singapore, the Republic of Korea, and China. Notably, institutions within this cluster, including Harvard University, the State University System of Florida, the Catholic University of Republic of Korea, and the Indian Institute of Technology System (iit system), exhibited close collaboration. The State University System of Florida demonstrated the highest collaborative effort among universities.

Conversely, the green cluster includes prominent universities from Saudi Arabia and India. Similar collaborative patterns were observed in the remaining clusters. Thus, the figure highlights the strong collaboration among institutions within the red cluster, with the State University System of Florida emerging as the most collaborative institution in diabetes research.

## 5. Summary of Performance Analysis

This section comprehensively examines various performance analyses through thematic evaluation and a three-field plot illustrating the interconnections among analytical approaches.

### 5.1. Thematic Evaluation

**Figure 15:**
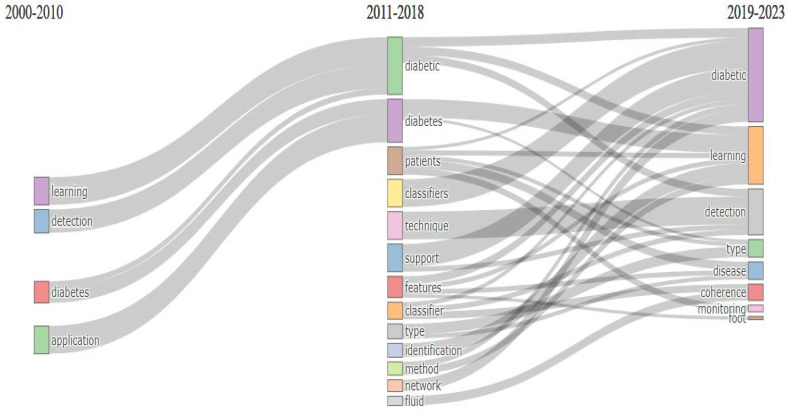
Thematic evaluation of diabetes detection.

The analysis of thematic evaluation is shown in Figure 15 through a Sankey diagram. A Sankey diagram is a graphical representation of information. In a Sankey diagram, the width of the line is proportional to the flow of information. Each box represents one theme, and this diagram illustrates the connection between different themes. From Figure 15, we can see that the terms “learning”, “detection”, “diabetes”, and “application”, were coined from 2000 to 2010, and the term “diabetes” was prevalent with researchers from 2011 to 2018. From 2019 to 2023, “detection” and “learning” still attracted researchers. They held the research interest in the same way from 2000 to 2023. At the beginning of 2011–2018, some other keywords were trendy for researchers, such as “diabetic”, “diabetes”, “patients”, “classifiers”, and “technique”. From 2019 to 2023, a few new themes appeared in the diabetes detection and classification research area. These are “disease”, “coherence”, and “monitoring”. The theme “diabetic” held the top spot with almost the same frequency from 2010 to 2023. However, from this analysis, we can also see that two themes remained constant with quite good frequency from 2000 to 2023: “learning” and “detection”.

### 5.2. Three-Field Plot

**Figure 16:**
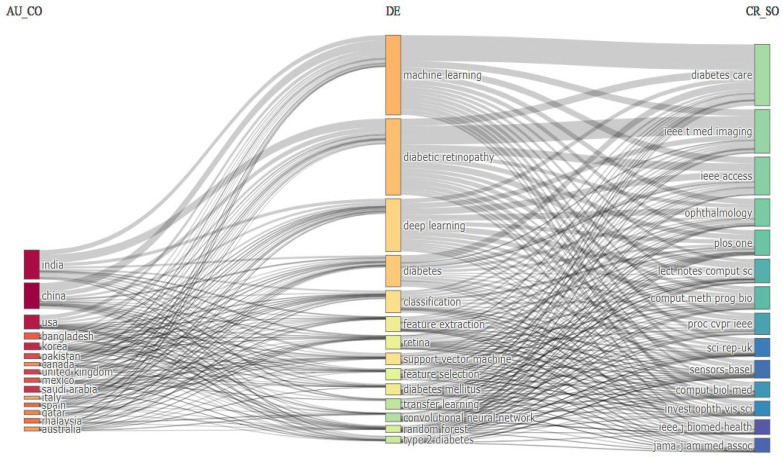
Three-field plot for diabetes detection.

An entire bibliometric analysis is possible in one figure in the three-field plot. It represents the vital relationship between different subjects for diabetes detection and classification. Figure 16 depicts the relationship between the 15 most productive countries, 15 frequently used keywords, and 15 primary journal sources. It represents which countries, keywords, and journal sources get more attention in this research area. For diabetes detection and classification, China and India published many papers. Next, the United States and Bangladesh, respectively, obtained the subsequent two positions. Figure 16 shows that “machine learning” was the most frequently used keyword in this research area. “Deep learning”, “diabetic retinopathy”, “diabetes”, and “classification” were also quite popular among diabetes researchers. From the field on the right, we can say that *Diabetes Care*, *IEEE T Med Imaging*, *IEEE Access*, and *Ophthalmology* were the primary journals. They published most of the papers on diabetes detection and classification.

## 6. Discussion

Diabetes mellitus is a significant global health concern, with a high prevalence and a high likelihood of increasing the risk of a few diseases like kidney failure, stroke, and heart attack. Considering this issue, and to provide comprehensive information in this context, our research focused on providing a systematic literature review based on the bibliometric method. Particularly, this section tries to summarize the findings of this research, along with a comparison of our work with similar literature-review-based works. Mainly, this section is divided into two subsections.

**Summarization of our findings:**

These research findings determine that India was the leading contributor, producing approximately 150 or more publications [31,32,33,34]. The most relevant author was Ali SHM, who contributed 10 documents on diabetes detection and classification [35]. Meanwhile, *IEEE Access* and *SENSORS* published the majority of the documents on diabetes detection and classification issues. *IEEE Access* had 24 published articles, and *SENSORS* had 18 articles. The trend analysis determined the most frequently used topics and keywords that attract researchers’ attention. “Machine learning”, “diabetic”, and “diabetes” were the most commonly used keywords for detecting and classifying diabetes. The papers’ citation analysis provided us with an overview of the most cited countries, authors, and sources in the field of diabetes from 2000 to 2023. The authors CORAM M, CUADROS J, GULSHAN V, KIM R, MADAMS T, and MEGA JL ([28,30,36,37]) received the highest number of citations for their significant research contribution to diabetes.

Along with the performance analysis, this paper also analyzed science mapping to comprehensively visualize and interpret the intricate relationships among various countries, institutions, and journals. Our focus on science mapping primarily explored the co-citation network of journals, the collaboration network of institutions, the collaboration network of countries, and the collaboration map delineating different continents and subcontinents. By constructing a collaboration network encompassing 15 countries, we observed China and the United States emerging as leaders with the most robust collaboration network. Lastly, the thematic evaluation and the three-field plots provided an overview of previous analyses of diabetes from 2000 to 2023. Along with this, the thematic evaluation showed that only “learning” and “detection” could maintain the researchers’ interest in the same way from 2000 to 2023. In 2023, the most popular topics were “diabetic”, “learning”, “detection”, diseases”, and “type”.

**Comparison with similar review papers:**

This subsection provides a comparative analysis by compiling information from several previously published similar review articles. This comparison is presented in Table 1.

All the articles presented in the table, including ours, have conducted bibliometric analyses on diabetes using various indices. However, this analysis followed the PRISMA framework, which enhanced the thoroughness of the study by including papers from 2000 to 2023. The bibliometric study highlighted recent trends and past status and correlated them. This paper also recognized the significant authors, countries, and journals.

## 7. Conclusions

This research conducted a bibliometric analysis of diabetes detection and classification, emphasizing “performance analysis” and “science mapping”, following the PRISMA framework. The findings of this paper will relatively help researchers to understand the development and impact of the research in the diabetes detection and classification arena. The analysis provides insights into the leading contributors, like countries, authors, affiliations, sources, trends, institutes, journals, and collaboration networks, in this particular research field. This will help the research community obtain information about the current landscape of this research area. The collaboration patterns were mapped between countries, keywords, and journals to identify strong partnerships and potential areas for future collaborations. The summary of the trend analysis showed that the research area of this particular field has shifted through the years. This will help to understand peoples’ current interest in this research field. This analysis will help people to find research gaps and will also help to decide the research agendas to address.

This research was conducted with only a single dataset, which was collected from the Web of Science (WOS) database. However, there are also a few other datasets available that have not been considered for this research, which could possibly narrow down the search space of relative studies. Due to the extensive length of the paper, we excluded a few performance analysis criteria, such as annual scientific production, average citations per year, Bradford’s law, references spectroscopy, and conceptual structure. Also, a few terms were not included in the search criteria, such as type 1 diabetes mellitus (DM), as the focus was on type 2 DM. Additionally, articles published in a wide range of journals and conferences might have been missed. Another limitation is this research did not investigate research methodological issues. In the future, these areas could be investigated. Additionally, this research did not focus on analyzing different literature review methods, like meta-analysis and conventional statistical analysis of these data.

## Figures and Tables

**Table 1:** Overview of key information from different similar review articles.

Author	Title	Keywords	Model/Methodology Used	Key Findings
Krishnamoorthy et al. [38]	Bibliometric analysis of Literature on Diabetes (1995–2004)	- Diabetes research - Bibliometric analysis, Bradford’s law - Relative growth rate	Bibliometric analysis using Bradford’s law, relative growth rate (RGR), and doubling time (Dt) analysis	- The United States was the country with the most contribution to diabetes research. - Research productivity conformed to Bradford’s law of scattering. - There was a decreasing relative growth rate (RGR) and increasing doubling time (Dt) over the years.
Jabali et al. [39]	A Bibliometric Analysis of Research Productivity on Diabetes Modeling and Artificial Pancreas 2001 to 2020	- Bibliometric - Diabetes, modeling, control - Glucose - Insulin dynamics	Bibliometric analysis using the Scopus database to explore research productivity in the field of diabetes modeling and control	- Leading countries, institutes, journals, articles, authorships, keywords, and research networks in the field were identified. Trends and gaps for future research in diabetes modeling and artificial pancreas development were highlighted.
Sweileh et al. [40]	Bibliometric Analysis of Diabetes Mellitus Research Output from Middle Eastern Arab Countries during the Period (1996–2012)	- Bibliometric, diabetes mellitus - Middle Eastern Arab countries	Scopus database used to analyze research output from Middle Eastern Arab countries in diabetes journals in 1996–2012, focusing on publication count, citation analysis, collaboration patterns, and journal impact factors	- Middle Eastern Arab countries contributed 0.75% to the global diabetes research output. - A significant increase in publications was observed after 2008. - The UAE was the top productive institution. - The total citations were 5565, with an h-index of 35.
Okaiyeto et al. [41]	Trends in Diabetes Research Outputs in South Africa over 30 years from 2010 to 2019|: A Bibliometric Analysis	- Bibliometric - Diabetes research - South Africa, analysis - Publication trends	Bibliometric analysis using the Scopus database to analyze research output in diabetes from South Africa, focusing on publication trends, author contributions, institutional output, and collaboration networks over a decade	- The study observed a consistent increase in diabetes research publications in South Africa between 2010 and 2019. - The University of Cape Town and Stellenbosch University were the leading institutions. - The most prolific author was Naomi Levitt [42]. International collaborations, particularly with the United States and the United Kingdom, were prominent. - The research focus shifted toward public health and epidemiology.
Gupta et al. [43]	Bibliometric Analysis of Diabetes Research in Relation to COVID-19	- Bibliometric - Diabetes - Global publications - COVID-19	Bibliometric analysis with the Scopus database using specific keywords; VOSviewer software used to visualize co-authorship and keyword co-occurrence networks for papers published between December 2019 and 6 January 2021	- There were a total of 762 publications on diabetes related to COVID-19 by 6 January 2021. - The United States, China, India, Italy, and the United Kingdom were the most productive countries. - Type 2 diabetes was most researched. - Pathophysiological studies were underrepresented.
Patel et al. [44]	Diabetes Prediction Using Machine Learning: A Bibliometric Analysis	- Bibliometric - Diabetes - Machine learning	Bibliometric analysis used, focusing on publication types, geographical data, keywords, and authors, primarily using data from Scopus and the Web of Science	- The most predominant sources were *Lecture Notes in Computer Science*, along with *PLOS One* and *Scientific Reports*. - The National Natural Science Foundation of China was the top funding organization, while the United States led in publications and citations.

## Data Availability

The raw data supporting the conclusions of this article will be made available by the authors upon request.
